# Investigating the role of the bacterial metabolite ectoine on immune regulation within the bovine female reproductive tract

**DOI:** 10.1093/jas/skag254

**Published:** 2026-08-20

**Authors:** Kaitlyn Weldon, Nathan Johnston, Kieran G Meade, Sean Fair

**Affiliations:** Department of Biological Sciences, Bernal Institute, Faculty of Science and Engineering, University of Limerick, Limerick V94 T9PX, Ireland; Department of Biological Sciences, Bernal Institute, Faculty of Science and Engineering, University of Limerick, Limerick V94 T9PX, Ireland; School of Agriculture and Food Science, University College Dublin, Dublin D04 V1W8, Ireland; Department of Biological Sciences, Bernal Institute, Faculty of Science and Engineering, University of Limerick, Limerick V94 T9PX, Ireland

**Keywords:** osmolyte, bull, sperm, immune system, anti-inflammatory

## Abstract

Ectoine, a compatible solute produced by extremophilic bacteria, is widely used for mucosal protection due to its cytoprotective and anti‑inflammatory properties. However, its effects within the female reproductive tract and its interaction with sperm‑associated immune responses remain unknown. This study provides the first assessment of ectoine’s immunomodulatory activity in bovine cervical and uterine tissues and its influence on sperm–polymorphonuclear neutrophil (PMN) interactions. Cervical and uterine explants from follicular‑phase heifers (*n* = 5) were pre‑incubated with 0, 0.5, 5, or 50 mM ectoine prior to stimulation with lipopolysaccharide (LPS) or sperm. Cytokine concentrations (IL‑8, IL‑6, and IL‑1β) were measured by ELISA, and sperm–PMN binding and sperm motility following PMN co‑incubation were evaluated. Baseline cytokine secretion was higher in uterine versus cervical explants (*P* < 0.05), indicating distinct tissue‑specific immune profiles. Ectoine alone did not alter basal cytokine secretion (*P* > 0.05). LPS increased IL‑8, IL‑6, and IL‑1β secretion from cervical explants and IL‑8 and IL‑1β in uterine explants (*P* < 0.05), and ectoine did not modify these responses (*P* > 0.05). Sperm elevated IL‑1β release from uterine explants (*P* < 0.01), while IL‑6 and IL‑8 remained unchanged; this response was also unaffected by ectoine (*P* > 0.05). Ectoine did not influence sperm–PMN binding or sperm motility in the presence of PMNs (*P* > 0.05). Collectively, this study provides the first reproductive‑tract safety evaluation of ectoine, demonstrating that it does not disrupt constitutive or stimulus‑induced inflammatory signaling nor interfere with sperm–immune interactions. These findings support ectoine’s safe incorporation into semen preservation and assisted reproductive technologies.

## Teaser

This study provides the first evaluation of ectoine’s immunological compatibility within the bovine female reproductive tract, demonstrating that it does not disrupt inflammatory responses or sperm–immune interactions. These findings support the safe use of ectoine in semen preservation and assisted reproductive technologies.

## Introduction

The immune response in the female reproductive tract is both dynamic and complex, and has yet to be fully characterized in livestock species. This complexity is shaped not only by the need to defend against pathogens but also by the reproductive demands placed on the tract during key physiological windows. One such window is estrus, during which hormonal fluctuations induce structural and functional changes—such as increased vascularity, uterine and cervical relaxation, and enhanced mucus secretion—that facilitate sperm transport and fertilization (Abreu et al. 2018). These changes render the tract vulnerable should microbial contamination occur, necessitating a highly selective and appropriately regulated immune response. This response must be capable of distinguishing between harmful pathogens and immunologically foreign sperm, while also tolerating the presence of a semi-allogeneic fetus (Akthar et al. 2021).

As haploid cells expressing paternal antigens, sperm are not immunologically inert; they can activate pattern recognition receptors (PRRs) within the female reproductive tract and directly influence local immune signaling (Ezz et al. 2019; Schjenken et al. 2021). The bovine cervix and uterus express a diverse repertoire of PRRs that detect both pathogen‑associated molecular patterns (PAMPs), such as lipopolysaccharide (LPS), and damage‑associated molecular patterns (DAMPs) released from host cells (Sheldon et al. 2017; Mansouri et al. 2025). Engagement of these receptors activates intracellular NF‑κB and MAPK signaling pathways, driving vascular changes, immune‑cell recruitment, and the production of key inflammatory mediators including chemokines and cytokines (Chastant and Saint-Dizier 2019). In cattle, sperm activate Toll‑like receptor 2 (TLR2) in uterine epithelial cells, stimulating the production of interleukin 8 (IL8), tumor necrosis factor alpha (TNFA) and interleukin 1B (IL1β) (Ezz et al. 2019). This acute response facilitates neutrophil recruitment and the clearance of excess or damaged sperm (Akthar et al. 2021), a phenomenon further supported by the rapid rise in uterine PMNs post‑insemination (Chastant and Saint-Dizier 2019).

Microbial exposure is a frequent and unavoidable feature of reproduction. Semen delivery—through either natural mating or artificial insemination—can introduce low‑grade bacterial contamination from the bull, equipment, or handling procedures (Bas et al. 2011). Similarly, calving is associated with substantial uterine microbial challenge and a corresponding inflammatory response (Sheldon et al. 2020). While acute inflammation is essential for microbial clearance and tissue repair, dysregulated or persistent inflammation can impair fertility. These challenges have intensified interest in identifying therapeutic strategies capable of modulating detrimental inflammation without compromising essential innate immune function.

Ectoine, a naturally occurring compatible solute synthesized by extremophilic microorganisms, has gained attention for its cytoprotective and immunomodulatory properties. Acting primarily through biophysical stabilization of membranes and macromolecules, ectoine protects epithelial tissues against osmotic, thermal, and inflammatory stress (Bethlehem and van Echten-Deckert 2021). Its beneficial effects are well documented across multiple mucosal systems, including respiratory, gastrointestinal, ocular, and dermatological tissues. Demonstrated efficacy has been shown in conditions such as allergic rhinitis (Werkhäuser et al. 2014), rhinosinusitis (Eichel et al. 2013), pharyngitis (Müller et al. 2016), dry eye disease (Chen et al. 2025), and inflammatory bowel disease (Abdel-Aziz et al. 2013).

Recently, our group was the first to demonstrate that ectoine enhances bovine sperm resilience to heat stress, osmotic imbalance, and prolonged exposure to cervical mucus (Weldon and Fair 2026). This work established ectoine as a promising candidate for semen preservation and assisted reproductive technology applications. Despite these advances, no studies have examined the effects of ectoine within the reproductive tract itself, and its interactions with local immune responses triggered by sperm or microbial cues remain completely uncharacterized. Addressing this knowledge gap is essential before ectoine can be safely integrated into reproductive technologies.

To address this gap, this study aimed to 1) assess the effect of ectoine on the non-stimulated immune response and compare basal immune profiles of the bovine cervix and uterus, 2) evaluate the anti-inflammatory effects of ectoine following stimulation with LPS, 3) evaluate the anti-inflammatory effects of ectoine following stimulation with sperm, and 4) assess the effect of ectoine on sperm–neutrophil interactions. These aims collectively deliver the first dedicated evaluation of ectoine’s immunological compatibility within the female reproductive tract, while generating new comparative data on cervical and uterine immune environments and establishing an ex vivo model for testing mucosal protectants in bovine fertility systems.

## Materials and methods

All procedures in this study were conducted in vitro using frozen bull semen samples collected under commercial conditions from an EU-licensed, AI-approved semen processing center following which semen straws were donated to research. Bovine female reproductive tracts were collected from an authorized local slaughterhouse. This study did not involve the use of living animals, therefore, ethical approval was not required.

### Chemicals and media

All chemicals were purchased from Sigma Aldrich (Arklow, Co Wicklow, Ireland) unless otherwise stated. Ectoine stock solutions were prepared in Roswell Park Memorial Institute (RPMI) and added directly to achieve final concentrations of 0.5, 5, or 50 mM. No further osmolarity adjustment was performed following ectoine supplementation.

### Semen collection, processing, and preparation

Semen was collected from mature Holstein Friesian bulls using an artificial vagina at a commercial stud. The ejaculate was fully extended in BullXcell (IMV Technologies) to a final concentration of 20 x 10^6^ sperm per 0.25 mL semen straw (IMV Technologies). Straws were filled, sealed and printed as per routine procedures. Straws were then cooled to 4 °C over 3 h and were frozen to −140 °C as follows: −5 °C per min from +4 °C to −10 °C, −40 °C per min from −10 °C to −100 °C and thereafter −20 °C per min from −100 °C to −140 °C in a programmable freezer (IMV Technologies), followed by submersion and storage in liquid nitrogen at −196 °C until use. Four straws from each ejaculate of each bull were assessed subjectively immediately post-thaw via standard microscopic techniques for total and gross motility (Murphy et al. 2018). Initial quality control cutoff values were total and gross motility of **≥**50% and a score of **≥**3, respectively. Ejaculates that passed the quality control were then donated to the project. Prior to use, semen straws were thawed at 37 °C for 30 s in a water bath, following which they were washed free from extender by centrifugation at 300 × *g* for 5 min, the sperm concentration was assessed using a hemocytometer and resuspended in media to achieve the desired sperm concentration.

### Media for washing and diluting sperm

The basal medium used for all experiments was a modified Tyrode’s Albumin Lactate Pyruvate (mTALP) medium supplemented with lactate and pyruvate and devoid of bicarbonate and bovine serum albumin (BSA) (Parrish et al. 1989). The mTALP consisted of 2 mM CaCl_2_, 3.1 mM KCl, 0.4 mM MgCl_2_, 125 mM NaCl, 0.3 mM NaH_2_PO_4_, 15 mM HEPES, 21.6 mM sodium lactate, and 1 mM sodium pyruvate. The medium was also supplemented with 0.5 mg/mL polyvinyl alcohol. Before using mTALP in experiments, the pH of the medium was adjusted to 7.3 with 1 N NaOH and to an osmolarity of 300 to 320 mOsm following which it was passed through a filter (0.22 μm pore size).

### Experimental design

#### Experiment 1

Comparison of basal immune profiles of the bovine cervix and uterus and assessment of the effect of ectoine on the constitutive immune response.

The objectives of this experiment were to characterize the basal immune response of uterine and cervical explants and to assess the effect of ectoine on the non-stimulated immune response. Reproductive tracts at the follicular phase of the estrous cycle were collected from non-pregnant nulliparous heifers (*n* = 5) at a commercial abattoir. Endometrial explants were recovered from the ipsilateral uterine horn and incubated for 24 h at 37 °C in 5% CO_2_ with 1) RPMI only (control) and 2) RPMI + 50 mM ectoine. The 24 h incubation period was selected in accordance with established bovine endometrial explant models, which have demonstrated that this duration maintains tissue viability while allowing sufficient accumulation of secreted inflammatory mediators for downstream analysis (Borges et al. 2012). A single high concentration of ectoine (50 mM) was assessed to determine whether it would elicit an inflammatory response in the cervical and uterine explants. Additional ectoine-only controls were not included, as Experiment 1 demonstrated that 50 mM ectoine did not alter constitutive cytokine secretion in cervical or uterine explants. Therefore, lower ectoine concentrations were considered unlikely to elicit an inflammatory response in the absence of LPS. The supernatant from the culture media was harvested and a panel of pro-inflammatory cytokines namely IL-8, interleukin 6 (IL-6) and IL-1β, were assessed for each treatment using an enzyme-linked immunosorbent assay (ELISA). These cytokines were selected because they are key mediators of innate immune responses in the female reproductive tract and play distinct roles in inflammation and sperm–immune interactions. IL-8 primarily functions as a potent chemoattractant for neutrophils, facilitating their migration to sites of inflammation (Proost et al. 1996). IL-6 is a multifunctional cytokine which plays a central role in immune response activation as well as other physiological functions including homeostatic immune and cellular processes like wound healing and cell repair (Aliyu et al. 2022). IL-1β is central proinflammatory cytokine involved in acute inflammation and amplification of the inflammatory cascade (Wijdan et al. 2025).

#### Experiment 2

The effect of ectoine against LPS-induced inflammation in cervical and uterine endometrial explants.

The objective of this experiment was to evaluate the effect of ectoine on LPS-induced inflammation in cervical and uterine explants. Reproductive tracts from non-pregnant, nulliparous heifers (*n* = 5) in the follicular phase of the estrous cycle were collected from a commercial abattoir. Cervical and uterine endometrial explants were recovered from the same animal and incubated for 3 h in one of the following treatments: 1) RPMI + 0 mM ectoine, 2) RPMI + 0.5 mM ectoine, 3) RPMI + 5 mM ectoine, 4) RPMI + 50 mM ectoine, and 5) RPMI only (control). Additional ectoine-only controls were not included in this experiment, as Experiment 1 demonstrated that the highest concentration tested (50 mM) did not alter constitutive cytokine secretion in cervical or uterine explants. Therefore, lower ectoine concentrations were considered unlikely to elicit an inflammatory response in the absence of LPS. After incubation, LPS (2 µg/mL) was added to all treatments except the RPMI-only control. The 3 h preincubation period was selected to allow sufficient tissue exposure and equilibration of ectoine prior to inflammatory challenge, ensuring that any potential immunomodulatory effects could be established before stimulation, while the subsequent LPS challenge (2 µg/mL) was selected to induce a robust and reproducible inflammatory response suitable for quantifying cytokine production (Donnellan et al. 2021). Supernatants were collected after a 24 h incubation and concentrations of the proinflammatory cytokines IL-8, IL-6, and IL-1β were quantified using an ELISA.

#### Experiment 3

The effect of ectoine against sperm-induced inflammation in uterine endometrial explants.

The objective of this experiment was to evaluate the anti-inflammatory potential of ectoine in response to sperm-induced inflammation using bovine uterine explants (*n* = 5 tracts). Explants were preincubated at 37 °C in 5% CO₂ for 3 h with ectoine at concentrations of 0, 0.5, 5, and 50 mM prior to sperm exposure. Following preincubation, frozen-thawed sperm from three bulls were washed, pooled to minimize individual bull variation and added to the explants at a final concentration of 5 × 10^6^ sperm per mL before incubation for an additional 6 h (Donnellan et al. 2021). LPS (2 µg/mL) served as a positive control, while explants incubated in RPMI only acted as the negative control. Supernatants were collected and levels of the proinflammatory cytokines IL-8, IL-6, and IL-1β were quantified to assess the inflammatory response under each treatment condition.

#### Experiment 4

The effect of ectoine on sperm–neutrophil interactions.

The objective of this experiment was to examine ectoine’s impact on sperm–neutrophil interactions. Six frozen-thawed semen straws from three bulls were pooled, washed, and diluted in phosphate buffered saline (PBS) containing 0.5% BSA. Sperm were preincubated for 1 h with ectoine at concentrations of 0, 0.5, 5, and 50 mM before being combined with PMNs at a 1:1 ratio. Sperm–PMN binding was assessed after 1 h of co-incubation at 37 °C in 5% CO₂ (Donnellan et al. 2021). Additionally, aliquots were collected at 0, 1, and 4 h for motility evaluation using computer assisted sperm analysis (CASA). Each treatment and time point was analyzed in duplicate (two technical replicates).

### Explant culture model

To investigate these effects in a physiologically relevant context, we employed an ex vivo explant model of bovine cervical and uterine tissues, which preserves native tissue architecture and immune responsiveness (Borges et al. 2012). Explant culture model was performed as described previously (Donnellan et al. 2021). Briefly, reproductive tracts from nulliparous heifers at the follicular stage were collected and uteri processed as described by Borges et al. (2012). The uterine horn ipsilateral to the ovary bearing the preovulatory follicle was opened longitudinally under sterile conditions. The endometrium was rinsed with Dulbecco’s PBS containing 1% antibiotic-antimycotic (ABAM), and intercaruncular biopsies were obtained using an 8 mm sterile biopsy punch. The endometrium was then dissected from the myometrium using sterile blades, washed twice in Hank’s balanced salt solution with 1% ABAM, and transferred to 24-well plates. Each well contained one explant in 1 mL RPMI medium supplemented with 5% heat-inactivated fetal bovine serum (FBS) and 1% ABAM. Explants were incubated endometrium-side up at 37 °C in 5% CO₂ for 1 h prior to treatment. Following stimulation with each treatment, tissues were snap frozen in liquid nitrogen and stored at −80 °C and culture supernatants were stored at −20 °C.

### ELISA

A series of sandwich ELISA assays were performed to quantify IL-8, IL-6, and IL-1β. The IL-8 ELISA was used to assess concentrations in cell supernatants as described by Cronin et al. (2015), using an ovine antibody, which has been shown to cross-react with cattle. The IL-6 and IL-1β ELISA kit (Thermo Fisher Scientific, Waltham, MA, USA) used a bovine specific antibody and was performed as per the manufacturer’s instructions.

### Polymorphonuclear neutrophil (PMN) isolation

Blood was collected from nulliparous heifers (*n* = 3) in estrus in 10 mL BD Vacutainer tubes (BD, Plymouth, UK), containing 18 mg of ethylenediaminetetraacetic acid. Blood samples were diluted 1:1 with PBS + EDTA and layered into sterile tubes over Histopaque-1077 (Sigma-Aldrich, Dorset, UK). Tubes were centrifuged at 800 × *g* for 40 min at 20 °C with no brake. The PMN layer was aspirated into sterile tubes and treated with red blood cell lysis buffer (Thermo Fisher Scientific) following the manufacturer’s instructions. After 10 min of gentle agitation, PBS was added to restore osmolarity and samples were centrifuged at 300 × *g* for 7 min at 20 °C. This wash step was repeated two additional times until the cell pellet appeared white. The final pellet was resuspended in 1 mL PBS containing 0.5% BSA. Purity was assessed by flow cytometry (CytoFLEX flow cytometer from Beckman Coulter [Labplan, Dublin, Ireland]) and only samples with >90% PMNs were used for subsequent treatments, which were initiated immediately after purity confirmation.

### Sperm–PMN binding assay

A final concentration of 2 × 10^6^ PMNs per mL and 20 × 10^6^ sperm per mL in 200 µL were incubated for 1 h at 37 °C and 5% CO_2_. Following incubation, samples were mixed to disassociate clumps and 10 µL was smeared on a slide and air dried. Slides were stained using Diff Quik staining according to manufacturer’s instructions. Sperm–PMN interaction was examined using brightfield and phase contrast microscopy (Olympus BX60) at 400× magnification, as described by Pini et al. (2018), where 200 PMNs were counted per slide and classified as free or bound to ≥1 sperm. For each slide, the number of sperm bound to a PMN was also recorded to calculate the number of bound sperm per PMN.

### CASA

Sperm samples were assessed using an IVOS II CASA system (IMV Technologies, L’Aigle, France), featuring a high-resolution internal camera with 1/1,000 s strobed illumination and 60 Hz frame rate, equipped with a Zeiss 10× negative phase-contrast objective (Zeiss 10x NH IVOS-II 160 nm) and temperature-regulated slide holder (37 °C). A 3 µL sample at 10 × 10^6^ sperm per mL in mTALP was loaded in a prewarmed 20-micron Leja chamber (IMV Technologies) and a minimum of 400 sperm per sample were assessed in a minimum of eight fields of view. For sperm detection, in order to ensure consistent, reliable evaluations, recommended factory-programmed CASA settings for bull sperm were used throughout (O’Meara et al. 2022). Parameters assessed included: total motility (%), progressive motility (%) and kinematic parameters including curvilinear velocity (VCL; μm/s), straight line velocity (VSL; μm/s), average path velocity (VAP; μm/s), straightness (STR; %), linearity (LIN; %) and the amplitude of lateral head movement (ALH; µm), as defined by Donnellan et al. (2022). Fixed motility settings were as follows: static = VAP < 5 µm/s and VSL < 3 µm/s; slow = VAP < 20 µm/s and VSL < 2 µm/s; progressive = STR ≥60% and VAP ≥ 40 (um/s). The cell size was between 6 and 70 µm^2^ and head minimum brightness was set to a minimum intensity of 190.

### Statistical analysis

The heifer was considered the experimental unit in all experiments. Data were analyzed using the Statistical Package for the Social Sciences (IBM SPSS for Windows, version 25.0). One-way ANOVA and repeated measures ANOVA were performed with Bonferroni post-hoc tests. Assumptions of normality and homogeneity of variances were assessed prior to one-way ANOVA using the Shapiro–Wilk test. Mauchly’s test of sphericity was used prior to repeated measures ANOVA. In Experiment 1, data were analyzed using a general linear model and cross-checked using a paired samples *t*-test. In Experiments 2 and 3, normally distributed cytokine data were analyzed using one-way ANOVA, while non-normally distributed data were analyzed using the Kruskal–Wallis nonparametric test. In Experiment 4, sperm–PMN binding was assessed using one-way ANOVA, and sperm motility was analyzed using repeated measures ANOVA (within-subject factor: time; between-subject factor: treatment). The level of statistical significance was set at *P* < 0.05.

## Results

### Experiment 1: comparison of basal immune profiles of the bovine cervix and uterus and assessment of the effect of ectoine on the constitutive immune response

**Figure 1:**
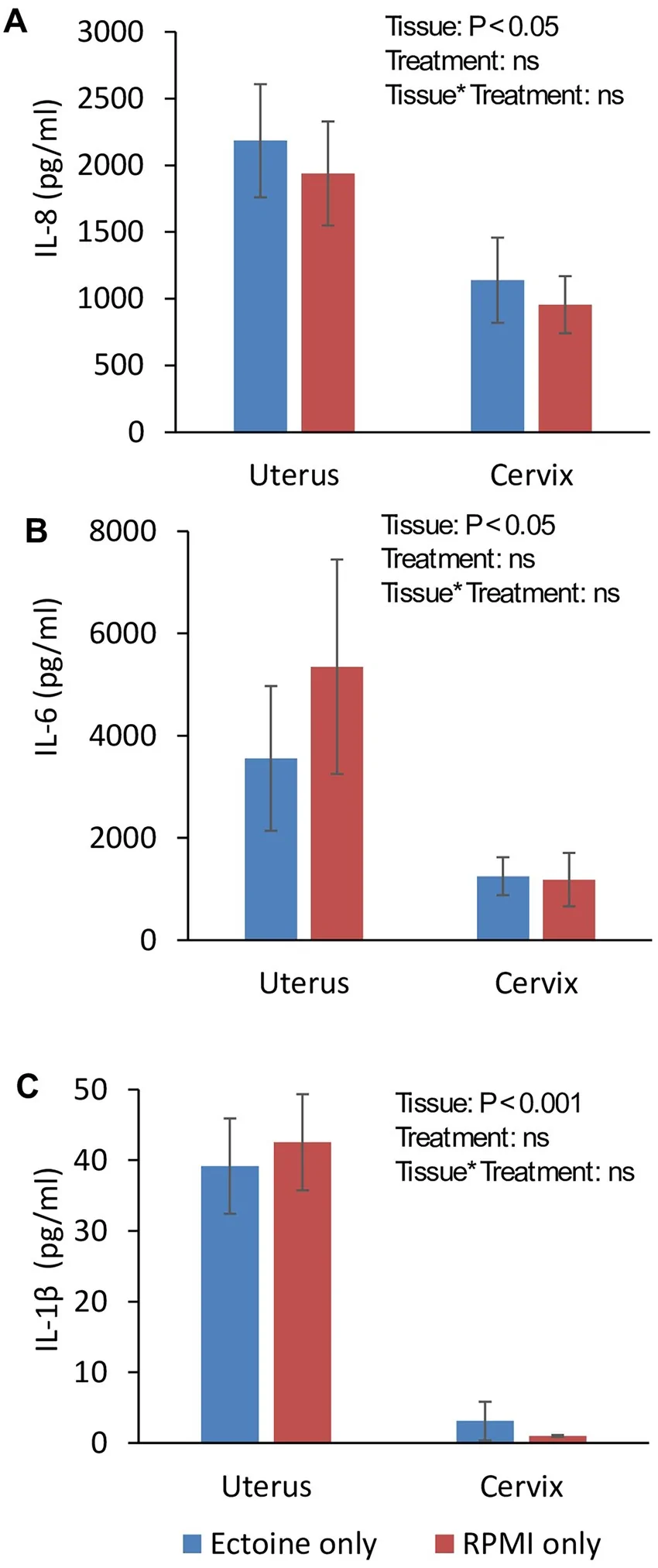
Concentrations of A) IL-8, B) IL-6, and C) IL-1β were assessed in uterine and cervical explants from the same heifers (*n* = 5) following a 24 h incubation with RPMI only (baseline control) or 50 mM ectoine. The ectoine only group assessed the effect of the molecule itself on basal cytokine production. Data are presented as mean ± SEM. ns = not significant.

Given that certain small molecules can act as immunostimulatory agents, this experiment aimed to determine whether ectoine alone, at the highest concentration tested in this study, in the absence of other stimuli, could elicit an immune response. Specifically, we compared cytokine production in ectoine-only treated samples to RPMI only controls to assess whether ectoine itself modulates baseline immune activity. There was no effect of ectoine on IL-8, IL-6, and IL-1β secretion from either the cervix or uterus (Figure 1; *P* > 0.05).

There was an effect of tissue type on constitutive IL-8, IL-6, and IL-1β production (*P* < 0.05). IL-8 secretion was approximately 2-fold higher in uterine compared to cervical explants in the absence of any inflammatory stimulus (*P* < 0.05). Similarly, IL-6 and IL-1β concentrations in culture supernatants were greater in uterine than in cervical explants (*P* < 0.05), indicating a tissue specific baseline immune profile (Figure 1).

### Experiment 2: the effect of ectoine against LPS-induced inflammation in cervical and uterine endometrial explants

**Figure 2:**
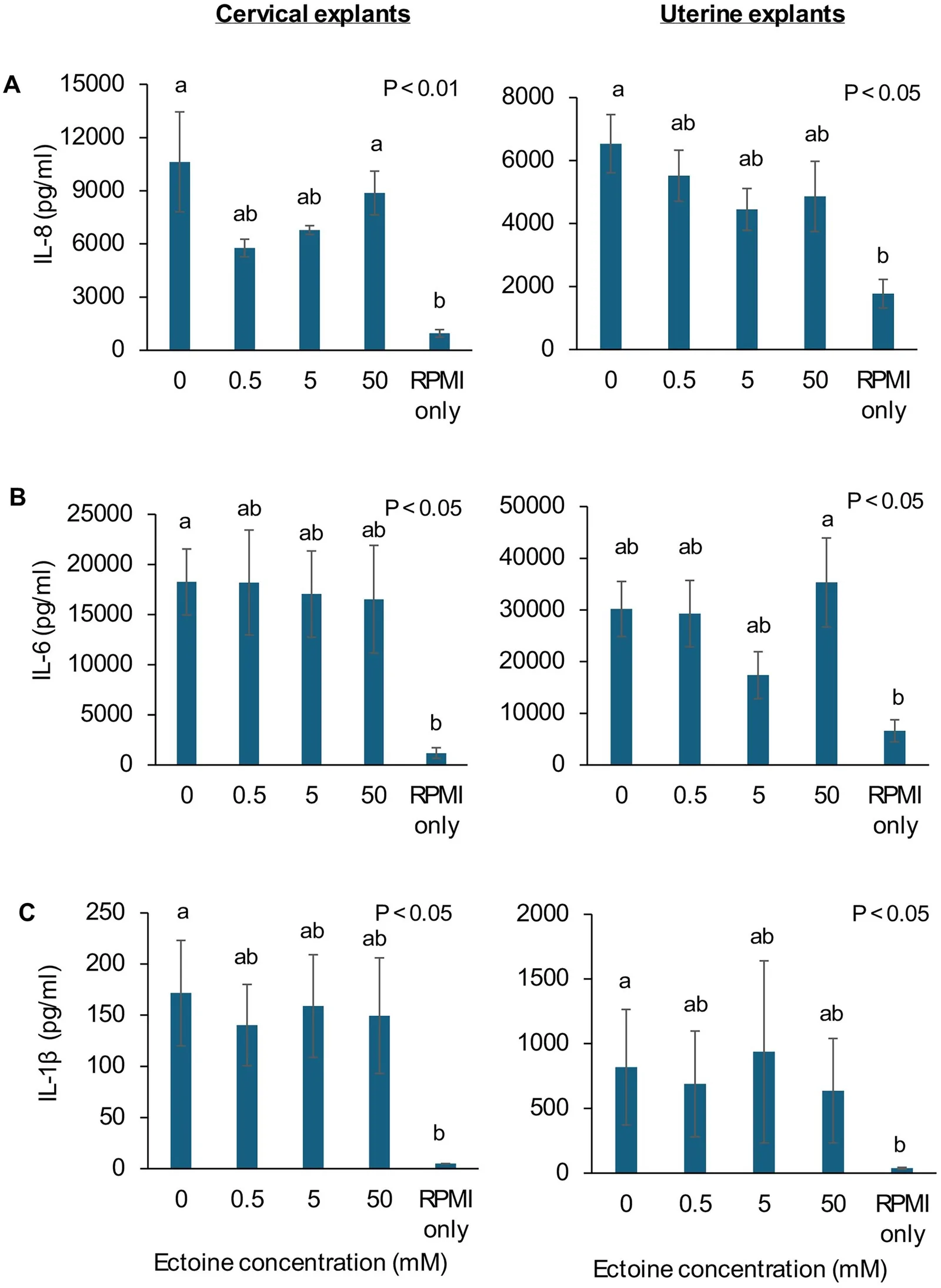
Concentrations of A) IL-8, B) IL-6, and C) IL-1β were assessed in culture supernatants of cervical explants (left column) and uterine endometrial explants (right column) exposed to lipopolysaccharide (LPS) (*n* = 5). Explants were recovered and preincubated with ectoine treatment (0, 0.5, 5, and 50 mM) for 3 h. After incubation, LPS (2 µg/mL) was added to all treatments except the RPMI-only control and explants were further incubated for 24 h. Data are presented as mean ± SEM. ^ab^Different superscripts differ significantly within each sub-figure (*P* < 0.05).

There was an effect of LPS treatment on IL-8, IL-6, and IL-1β concentrations in both cervical and uterine explant culture supernatants (Figure 2; *P* < 0.05). In cervical tissue, IL-8 levels increased approximately 11-fold following LPS stimulation compared to RPMI-only controls (*P* < 0.01), while uterine explants showed a 4-fold increase in IL-8 levels (*P* < 0.05). Across both tissues, ectoine treatment (0.5, 5, and 50 mM) did not alter IL-8 concentrations (*P* > 0.05).

Following LPS exposure, IL-6 levels increased in cervical explant supernatants (*P* < 0.05), but not in uterine explants (Figure 2; *P* > 0.05). In both tissues, IL-6 concentrations remained consistent across ectoine treatment groups (*P* > 0.05). LPS stimulation increased IL-1β secretion from both cervical and uterine explants (*P* < 0.05). Ectoine treatment did not affect IL-1β concentrations in either tissue (*P* > 0.05).

### Experiment 3: the effect of ectoine against sperm-induced inflammation in uterine endometrial explants

**Figure 3:**
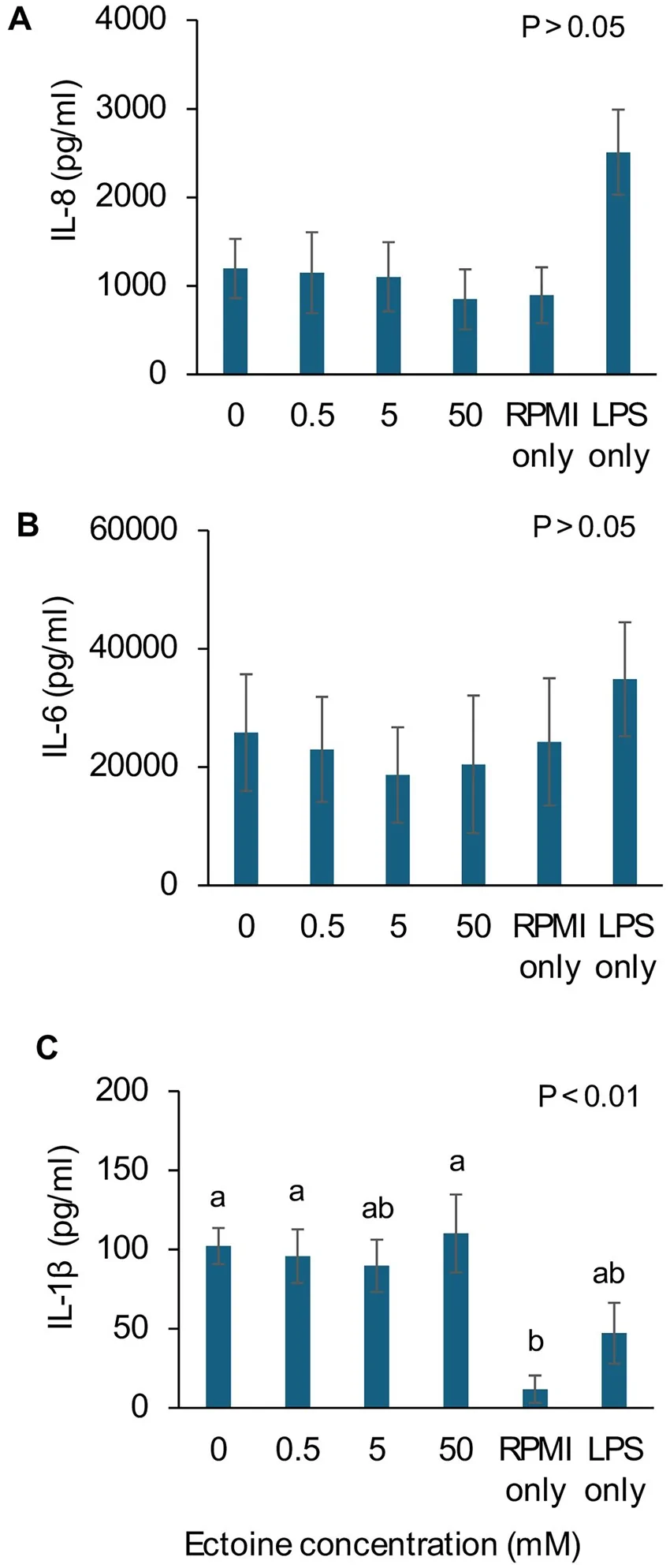
Concentrations of A) IL-8, B) IL-6, and C) IL-1β were measured in culture supernatants of uterine explants exposed to sperm (*n* = 5). Explants were preincubated for 3 h with ectoine at concentrations of 0, 0.5, 5, and 50 mM prior to sperm exposure. Following preincubation, sperm was added to the explants at a final concentration of 5 × 10^6^ sperm per mL and incubated for an additional 6 h. LPS (2 µg/mL) served as a positive control, while explants incubated in RPMI only acted as the negative control. Data are presented as mean ± SEM. ^ab^Different superscripts differ significantly within each sub-figure (*P* < 0.05).

Sperm exposure did not affect IL-6 or IL-8 concentrations; however, IL-1β levels were elevated compared to the control (Figure 3; *P* < 0.05). Preincubation of uterine endometrial explants with ectoine at concentrations of 0, 0.5, 5, and 50 mM prior to sperm exposure had no effect on IL-8, IL-6, and IL-1β concentrations (Figure 3; *P* > 0.05). Notably, the 5 mM ectoine group showed no difference in IL-1β levels compared to the other ectoine-treated groups but was also statistically similar to the RPMI-only control (*P* > 0.05).

### Experiment 4: the effect of ectoine on sperm–neutrophil interactions

**Figure 4:**
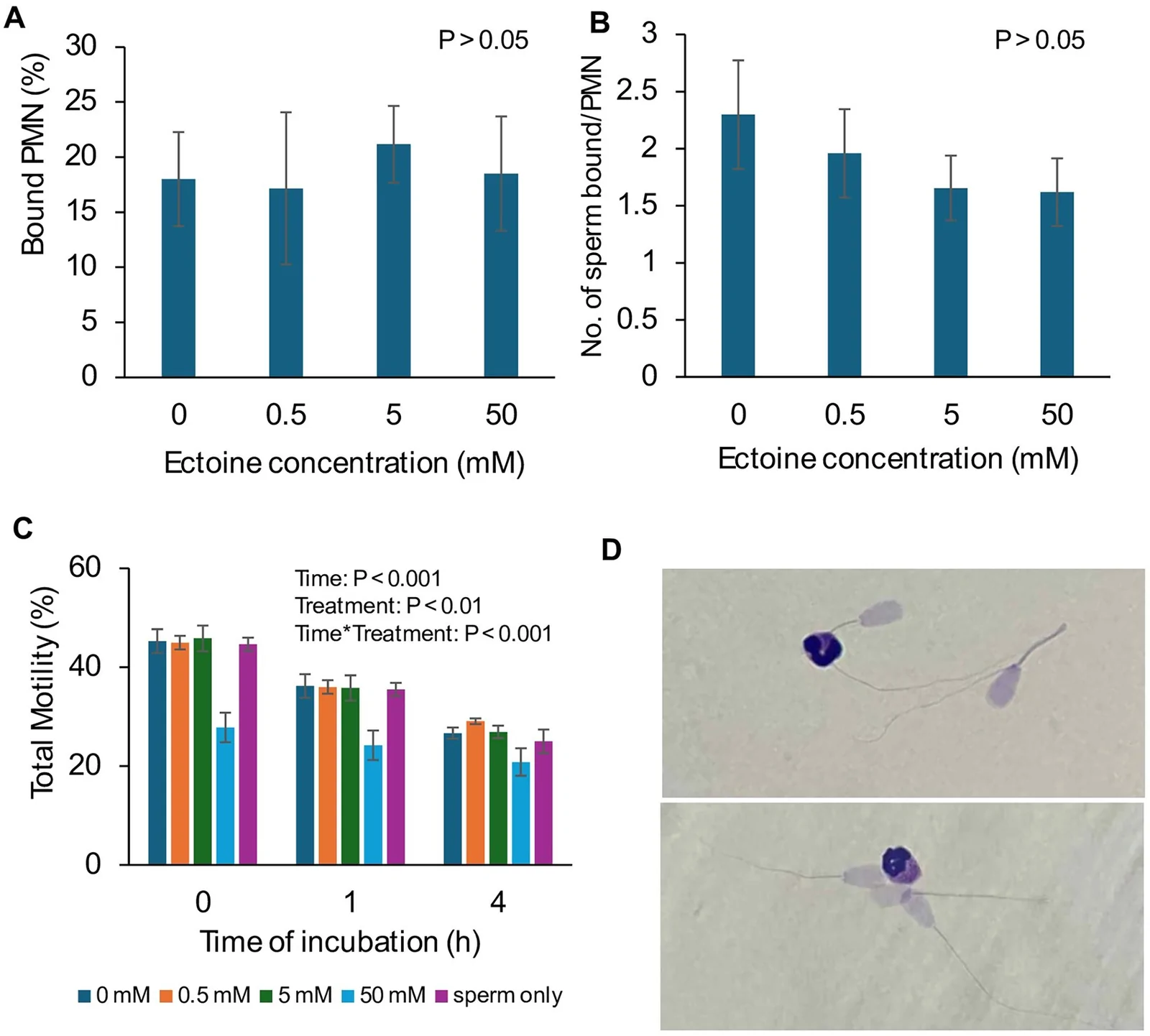
The effect of ectoine on sperm–neutrophil interactions (*n* = 3). A) percentage of bound PMNs (≥1 sperm bound) from sperm preincubated with ectoine (0–50 mM), B) number of bound sperm per PMN, C) total motility (%) of ectoine treated sperm incubated with PMNs, measured at 0, 1, and 4 h, D) representative images showing (top) a PMN bound to the midpiece of one sperm and (bottom) a PMN directly bound to two sperm heads. A sperm was classified as bound to a PMN if the head or mid-piece region was in direct contact with the PMN. Data are presented as mean ± SEM.

There was no effect of treatment on the percentage of PMNs bound to ≥1 sperm (Figure 4; *P* > 0.05). On average, each PMN bound 2.3 ± 0.47 sperm, and this was not influenced by ectoine treatment (*P* > 0.05). An effect of time was observed on total sperm motility, with motility declining across all treatments over the 4-h incubation period (*P* < 0.001). Notably, sperm treated with 50 mM ectoine exhibited reduced motility at both 0 and 1 h compared to the lower ectoine concentrations (*P* < 0.05) and this had no effect on sperm–PMN binding. However, sperm treated with 0, 0.5, or 5 mM ectoine and co-incubated with PMNs maintained total motility levels comparable to the sperm-only control (Figure 4; *P* > 0.05).

## Discussion

This study provides the first comprehensive assessment of ectoine’s immunological compatibility with the bovine female reproductive tract and its influence on inflammatory responses triggered by microbial or sperm exposure. Using ex vivo cervical and uterine explants, the results show that ectoine does not stimulate basal inflammatory activity, does not dampen LPS- or sperm-induced cytokine production, and does not interfere with sperm–neutrophil interactions. These findings collectively indicate that ectoine is immunologically inert within reproductive tissues, supporting its safe application in reproductive technologies.

A key novel observation from this work is the clear distinction in baseline cytokine profiles between cervical and uterine tissues. Across all measured cytokines, IL-8, IL-6, and IL-1β-uterine explants displayed consistently higher constitutive cytokine secretion than cervical explants. This aligns with the known functional immunology of the bovine reproductive tract, where the lower tract serves as a relatively stable barrier against environmental pathogens, while the upper tract undergoes cyclical fluctuations in immune-cell composition during the estrous cycle to support fertilization and early pregnancy (Kuni et al. 2025). The high leukocyte density reported in the bovine endometrium is consistent with the elevated cytokine production observed here. These findings contribute new comparative data on cervical versus uterine immune responses, an area that remains poorly characterized in livestock, and provide an important reference point for future studies evaluating mucosal immune responses in reproductive tissues.

Ectoine did not induce cytokine production under basal conditions, even at the highest concentration tested. This confirms that ectoine does not act as an immunostimulatory molecule within reproductive tissues and is consistent with findings in other systems, including human keratinocytes, in which the sole ectoine treatment did not modify the level of expression of inflammatory genes examined including *IL1A*, *IL6*, *IL8*, and *TNFA* (Buommino et al. 2005). Similarly, in a rat model of lung inflammation, ectoine did not induce increased neutrophil recruitment when administered in the absence of an inflammatory challenge (Sydlik et al. 2009). The absence of any proinflammatory effect is a critical safety indicator, given that some small molecules can trigger PRR-mediated immune activation. These results demonstrate that ectoine maintains immune quiescence in both cervical and uterine tissues, reinforcing its classification as a biologically compatible osmoprotectant.

As expected, LPS stimulation induced significant inflammatory responses in both cervical and uterine explants, with increased secretion of IL-8 and IL-1β from both tissues and IL-6 in the cervix. These results corroborate earlier reproductive studies demonstrating LPS-driven expression of IL8, IL1A, IL1β, and TNFA in bovine endometrial cells and explants (Ezz et al. 2019; Donnellan et al. 2021). Notably, ectoine did not alter these LPS-induced responses. Although subtle reductions in IL-8 and IL-6 were observed in some uterine explant replicates following 5 mM ectoine treatment, these were not statistically significant. A similar pattern has been reported in an in vivo respiratory models, where ectoine reduced nanoparticle-induced inflammation but had limited effect on LPS-driven responses (Sydlik et al. 2009). Together, these results suggest that while ectoine may modulate certain stress-induced pathways, it does not interfere with PAMP-mediated inflammatory signaling, an important consideration for preserving essential innate immune function in the reproductive tract.

Because sperm-induced inflammation occurs predominantly in the uterus, particularly following artificial insemination, we focused on sperm–tissue interactions in uterine explants. Sperm exposure selectively increased IL-1β, with no effect on IL-6 or IL-8. This finding aligns with Donnellan et al. (2021), who reported unchanged *IL6* gene expression alongside increased *IL1β* expression and protein levels in bovine uterine explants following sperm exposure. Complementary evidence from Akthar et al. (2023) further supports this, showing that sperm interacts with the uterine gland, triggering the expression of inflammatory mediators including *IL1β.* Notably, both studies also observed increased *IL8* expression, which contrasts with the current findings. This discrepancy may reflect the transient and weak nature of sperm-induced inflammation in bovine endometrium (Mansouri et al. 2023). Inter-animal variation in immune responsiveness, which can be more pronounced in ex vivo systems, may also contribute to the observed differences. Importantly, ectoine pretreatment did not alter the sperm-induced IL-1β response, further supporting its lack of interference with physiological sperm–immune signaling.

A critical component of post-insemination immunity is the interaction between sperm and PMNs. Neutrophil recruitment to the uterus facilitates clearance of excess or damaged sperm without compromising fertilization potential (Akthar et al. 2021). Here, ectoine did not alter the proportion of PMNs bound to sperm nor the number of sperm bound per PMN. Notably, although sperm treated with 50 mM ectoine exhibited reduced motility during incubation, this had no effect on sperm–PMN binding. This finding is consistent with our previous study, in which 50 mM ectoine was associated with reduced sperm velocity parameters and lower hyperactivation, suggesting that this concentration may be suboptimal compared with lower doses (Weldon et al. 2026). However, the lack of any effect on sperm–PMN binding indicates that ectoine does not impair neutrophil recognition or engagement with sperm. Combined with the absence of ectoine-induced cytokine release, this reinforces the conclusion that ectoine is immunologically neutral within the cervix and the uterus. Moreover, the stability of sperm motility in the presence of PMNs suggests that the explant-conditioned media used here did not impose additional detrimental effects on sperm function.

Overall, this study represents the first evaluation of ectoine within the context of reproductive-tract immunity. Across all experiments, ectoine demonstrated no proinflammatory activity, no suppression of pathogen- or sperm-induced cytokine responses, and no disruption of sperm–PMN interactions. Importantly, lower concentrations (0.5–5 mM) maintained sperm function while remaining immunologically compatible with reproductive tissues. When interpreted alongside our previous work demonstrating that ectoine enhances bovine sperm resilience to thermal and osmotic stress and prolonged exposure to mucus (Weldon et al. 2026; Weldon and Fair 2026), these findings strongly support ectoine’s biocompatibility with both sperm and reproductive tissues and suggest that concentrations within this range may be the most suitable for future reproductive applications. Collectively, this evidence positions ectoine as a promising candidate for inclusion in semen preservation strategies and provides an essential foundation for future studies exploring its safe application in reproductive biotechnology.

## Abbreviations

ABAM: antibiotic-antimycotic
ALH: amplitude of lateral head movement
BSA: bovine serum albumin
CASA: computer-assisted sperm analysis
DAMPs: damage-associated molecular patterns
ELISA: enzyme-linked immunosorbent assay
FBS: fetal bovine serum
IL-1β: interleukin-1 beta
IL-6: interleukin-6
IL-8: interleukin-8
LPS: lipopolysaccharide
MAPK: mitogen-activated protein kinase
mTALP: modified Tyrode’s albumin lactate pyruvate
NF-κB: nuclear factor kappa B
PAMPs: pathogen-associated molecular patterns
PBS: phosphate-buffered saline
PMN: polymorphonuclear neutrophil
PRR: pattern recognition receptor
RPMI: Roswell Park Memorial Institute medium
TLR: Toll-like receptor
TNFA: tumor necrosis factor alpha
VAP: average path velocity
VCL: curvilinear velocity
VSL: straight-line velocity
